# Cholesterol added prior to vitrification on the cryotolerance of immature and *in vitro* matured bovine oocytes

**DOI:** 10.1371/journal.pone.0184714

**Published:** 2017-09-14

**Authors:** Núria Arcarons, Roser Morató, Meritxell Vendrell, Marc Yeste, Manel López-Bejar, Kosala Rajapaksha, Muhammad Anzar, Teresa Mogas

**Affiliations:** 1 Department of Animal Medicine and Surgery, University Autonomous of Barcelona, Cerdanyola del Vallès, Spain; 2 Department of Animal Health and Anatomy, University Autonomous of Barcelona, Cerdanyola del Vallès, Spain; 3 Institute of Food and Agricultural Technology, University of Girona, Girona, Spain; 4 Agriculture and Agri-Food Canada, Saskatoon Research and Development Centre and Western College of Veterinary Medicine, University of Saskatchewan, Saskatoon, Canada; Hull York Medical School, UNITED KINGDOM

## Abstract

This study examines whether incorporating cholesterol-loaded methyl-β-cyclodextrin (CLC) in the bovine oocyte plasma membrane improves oocyte tolerance to vitrification. *In vitro* matured oocytes were incubated with 2 mg/ml BODIPY-labeled CLC for different time intervals in FCS or PVA supplemented medium or exposed to different CLC concentrations to examine the subcellular localization of cholesterol by confocal microscopy live-cell imaging. Subsequently, the effects of optimized CLC concentrations and incubation times prior to vitrification on early embryo development were assessed. Then, we evaluated the effects of pretreatment with 2 mg/ml CLC for 30 min before the vitrification of immature (GV) and *in vitro* matured (MII) oocytes on developmental competence and gene expression. Our results indicate a high plasma membrane labeling intensity after 30 min of incubation with 2 mg/ml CLC for 30 min, regardless of the holding medium used. When oocytes were incubated with 1 mg/ml, 2 mg/ml and 3 mg/ml of CLC, intense labeling was observed at the plasma membrane after 40, 30 and 20 min, respectively. CLC pre-treatment before the vitrification of bovine oocytes did not affect subsequent cleavage and embryo development rates irrespective of CLC concentrations, incubation times or meiotic stage. However, pretreatment seems to improve the quality of embryos derived from vitrified oocytes, mainly when oocytes were vitrified at the GV stage.

## Introduction

Widespread use of animal oocytes for procedures such as *in vitro* embryo production, nuclear transfer or gene banking has dramatically increased interest in oocyte cryopreservation in the agricultural and scientific communities [1]. The practical benefits of vitrification to preserve bovine oocytes are nevertheless limited since vitrified oocytes show impaired *in vitro* maturation and early embryo development.

During oocyte cryopreservation, cooling and osmotic stress can cause irreversible damage to membrane integrity [2, 3]. Oocytes undergo substantial volume changes due to water and cryoprotectant movement during cryopreservation (reviewed in: [4]). This suggests that cells with more flexible membranes permeable to water and cryoprotectants are likely to suffer less damage that those with more rigid, less permeable membranes. The incorporation of cholesterol in the plasma membrane should enhance membrane fluidity and permeability at low temperatures, and thereby increase oocyte tolerance to cryopreservation.

Cyclodextrins (CDs) are cyclic oligosaccharides consisting of five or more α-D-glucopyranose residues linked by α-1,4 glucosidic bonds that have a hydrophobic center capable of integrating lipids. The high affinity of βCDs for cholesterol, besides conferring the capacity to remove cholesterol from biological membranes, also enables the formation of cholesterol inclusion complexes that donate cholesterol to the membrane. The efficiency of cholesterol transfer from βCD inclusion complexes to biological membranes depends on the βCD:cholesterol molar ratio, βCD-cholesterol concentration, and treatment duration [5, 6].

In prior work in oocytes it was observed that the co-incubation of bovine immature [7] or *in vitro-*matured [8] oocytes with βCDs loaded with cholesterol (CLC) improved the nuclear maturation of oocytes after vitrification, but did not benefit embryo development to the blastocyst stage. These authors attributed the lack of effect of CLC treatment in improving cryotolerance that cholesterol was preferentially transferred to lipids or proteins present in the fetal calf serum (FCS) avoiding its incorporation into the oocyte. When examining the incorporation of cholesterol in the oocyte plasma membrane after incubation with CLC, Horvath and Seidel [8] and Jiménez-Trigos et al [9] observed that cholesterol was transported through the cumulus layers and the zona pellucida into the oocyte. However, little information was provided regarding the exact localization of cholesterol within the oocyte or how much cholesterol entered the cell. Boron-dipyrromethene (BODIPY)-cholesterol BODIPY-cholesterol has been used as a cholesterol probe in model membranes and in trafficking studies in living cells [10, 11]. Through *in vivo* time-lapse analysis, the time-point at which this probe was located at the plasma membrane of mouse oocytes has been identified [12]. We therefore hypothesized that this new fluorescent cholesterol probe would be useful to determine the dose and incubation time at which cholesterol locates mainly at the plasma membrane of bovine oocytes before vitrification.

Previous studies have shown that basic cryobiological differences exist between immature and mature oocytes. Mature metaphase II-stage (MII) oocytes can be difficult to cryopreserve, mainly because of the presence of the meiotic spindle and chromosome configuration. By contrast, in immature germinal vesicle-stage (GV) oocytes, spindle depolymerization during cryopreservation is avoided but oocytes at GV stage aremore sensitive to osmotic stress than MII oocytes [13]. Therefore, the improvement in membrane fluidity conferred by membrane cholesterol enrichment has strong potential to enhance tolerance of GV oocytes to vitrification.

The present study was designed to examine whether exposure of bovine oocytes to CLC before vitrification/warming could improve their cryotolerance and embryo development after *in vitro* fertilization. In a first set of experiments, we characterized intracellular trafficking and localization of fluorescently-labeled cholesterol in *in vitro* matured oocytes incubated with CLC either in FCS or PVA supplemented medium and assessed their effect on early embryo development after vitrification/warming. In a second set of experiments, different concentrations of CLC were compared in terms of the subcellular localization of fluorescently-labeled cholesterol. Subsequently, the effects of optimized CLC concentrations and incubation times prior to vitrification on early embryo development were assessed. Finally, immature or *in vitro* matured oocytes were vitrified after 30 min of incubation with 2 mg/ml CLC, and then fertilized and cultured to determine early embryo development and quality, and at the level of expression of specific genes that are potentially important in embryo survival. The expression patterns of genes involved in apoptosis (*BAX*), lipid metabolism (*CYP51*), imprinting (*DNMT3A*, *IGF2R*, *UBE2A*), heat (*HSPA1A*) and oxidative stress (*MnSOD*) were determined on Day 5 morulae by RT-qPCR.

## Material and methods

### Chemicals and supplies

All chemicals and reagents were purchased from Sigma Chemical Co. (St. Louis, MO, USA) unless otherwise stated.

### Bovine oocyte collection and *in vitro* maturation

The methods used for the *in vitro* maturation of the bovine oocytes have been described elsewhere [14]. Briefly, bovine ovaries were collected at slaughter from a local abattoir (Escorxador Sabadell, S.A., Sabadell, Spain) and transported to the laboratory in phosphate buffered saline (PBS) at 35–37°C. Cumulus oocyte complexes (COCs) were obtained by aspirating 2–10 mm follicles. Only COCs with three or more layers of cumulus cells and a homogeneous cytoplasm were selected to be matured *in vitro*. After three washes in modified PBS (PBS supplemented with 36 mg/ml pyruvate, 50 mg/ml gentamicin and 0.5 mg/ml bovine serum albumin (BSA)), groups of up to 50 COCs were placed in 500 μl of maturation medium in four-well plates and cultured for 24 h at 38.5°C in a 5% CO_2_ humidified air atmosphere. The maturation medium was comprised of TCM-199 supplemented with 10% (v/v) FCS, 10 μg/ml epidermal growth factor and 50 mg/ml gentamicin.

### Cholesterol-loaded methyl-β-cyclodextrin (CLC)

The method described by Purdy and Graham [15] was used to prepare CLC. Briefly, 1 g of methyl-β-cyclodextrin was dissolved in 2 ml of methanol by vortexing. Separately, 200 mg cholesterol was dissolved in 1 ml of chloroform. A 0.45 ml aliquot of the cholesterol solution was then gently mixed with the 2 ml of cyclodextrin solution until combined to form a clear solution. The solvents were removed from the mixture with a stream of nitrogen gas and the crystals obtained were dried for 24 h, stored at room temperature, and designated cholesterol-loaded methyl-β-cyclodextrin (CLC).

### Cholesterol imaging in living bovine oocytes treated with CLC

To determine cholesterol trafficking, oocytes were incubated together with different concentrations of CLC (see experimental design) complexed with 1 μM BODIPY-cholesterol (BPY-Chol. Avanti Polar Lipids, Alabama, USA) [12, 16] at 38.5°C and 5% CO_2_ in air. BPY-Chol was visualized in living oocytes using a confocal microscope Leica TCS SP5 (Leica Microsystems GmbH, Mannheim, Germany). BPY-Chol was excited with a 488 nm argon laser with fluorescence emission detected in the 500–620 nm range using a HyD detector to identify the intracellular location of the fluorescent probe after incubation at different chase times (see experimental design).

The fluorescence intensity of plasma membrane and cytoplasm was quantified using ImageJ Software. After subtracting the background, cytoplasm mean intensity was measured by manually outlining the area corresponding to the oocyte cytoplasm, excluding the plasma membrane. A 55-pixel band was then created to surround the plasma membrane and mean fluorescence intensity for the plasma membrane was determined. The images were segmented into foreground and background by setting a threshold that was the same for all samples.

### Oocyte vitrification and warming

#### Vitrification protocol

The vitrification/warming procedures employed were essentially as described by Morató *et al*. [17]. The holding medium (HM) used to formulate the vitrification-warming solutions consisted of HEPES-TCM-199 supplemented with 20% (v/v) FCS. All steps were performed under a laminar flow hood heated to 38.5°C using a stereomicroscope to visualize each step. Partially denuded oocytes were transferred into equilibration solution (ES) consisting of 7.5% (v/v) ethylene glycol (EG) and 7.5% (v/v) dimethylsulfoxide (DMSO) in HM for 10 min. Subsequently, oocytes were moved to the vitrification solution (VS) containing 15% (v/v) DMSO, 15% (v/v) EG and 0.5 M sucrose dissolved in HM. After incubating for 30–40 s, oocytes were loaded onto a cryotop. Almost all the solution was removed to leave only a thin layer covering the oocytes and the cryotop plunged into liquid nitrogen. The entire process from exposure to VS to plunging in liquid nitrogen was completed in 90 seconds. When synthetic medium was required (see experimental design), 6% polyvinylpyrrolidone (PVP) (w/v) and 1 mg/ml fetuin were added to the equilibration and vitrification solutions instead of FCS.

#### Warming protocol

Vitrified oocytes were warmed by directly immersing the cryotop into the warming solution containing 1 M sucrose dissolved in HM for 1 min. Next, the recovered oocytes were transferred to the dilution solution containing 0.5 M sucrose dissolved in HM for 3 min. The oocytes were then incubated in HM for 5 min. After a final rinse in HM for 1 min, oocytes were transferred to the maturation medium and allowed to complete their *in vitro* maturation.

### *In vitro* fertilization and embryo culture

*In vitro* matured oocytes (fresh and vitrified/warmed) were *in vitro* fertilized at 38.5°C in a 5% CO_2_ atmosphere. Frozen–thawed spermatozoa from Asturian bulls (ASEAVA, Llanera, Asturias, Spain) of proven fertility were used in all the experimental procedures. Motile spermatozoa were obtained by centrifuging frozen–thawed sperm on a discontinuous gradient (Bovipure, Nidacon International, Gothenburg, Sweden) for 10 min at 100×g at room temperature. Viable spermatozoa collected from the bottom were washed (Boviwash, Nidacon International, Gothenburg, Sweden) and pelleted by centrifugation at 100×g for 5 min. Spermatozoa were counted in a Neubauer hemocytometer and diluted in an appropriate volume of fertilization medium (Tyrode’s medium supplemented with 25 mM bicarbonate, 22 mM Na-lactate, 1 mM Na-pyruvate, 6 mg/ml fatty acid-free BSA and 10 mg/ml heparin–sodium salt (Calbiochem, Darmstadt, Germany)) to a final concentration of 1 × 10^6^ spermatozoa/ml. 100-μl droplets of diluted sperm were prepared under mineral oil and 20 to 25 oocytes/droplet co-incubated at 38.5°C, in a 5% CO_2_ high humidity atmosphere.

After 18–20 h, presumptive zygotes were stripped of remaining cumulus cells by gentle vortexing after which they were cultured in 100-μl drops of synthetic oviductal fluid (SOF) [18] containing FCS (5%, v/v) under mineral oil at 38.5°C in 5% CO_2_, 5% O_2_, 90% N_2_ for 8 days.

Cleavage rates were recorded at 48 h post-insemination (hpi) and the number of blastocysts was determined on Days 7 and 8 post-insemination (pi). Day 8 embryos were classified according to the degree of blastocele expansion into three groups according to Morató *et al*. [19]: (1) non-expanded blastocysts, in which the blastocele volume was less than one-half of the total volume of the blastocyst; (2) expanded blastocysts, in which the blastocele volume was more than one-half of the total volume of the blastocyst; (3) hatched or hatching blastocysts, in which the expanded blastocyst was without a zona pellucida or had an opened zona pellucida.

### RNA extraction and real-time quantitative (rt)-PCR

Total RNA was extracted from Day-5 morulae using the Picopure RNA extraction Kit (Thermo Fisher Scientific Inc, Waltham, MA, USA) following the manufacturer’s instructions. Morulae harvested from each experimental group were pooled in groups (10 morulae) and washed three times in Dulbecco’s-PBS at 38.5°C. Each pool was added to 100 μl of extraction buffer, snap frozen in liquid nitrogen, and stored at– 80°C until RNA isolation. A total of five pools for each experimental group were analyzed.

For RNA extraction, samples were vortexed for 15 s and incubated with lysis solution at 42°C for 30 min. The samples were then centrifuged at 3,000×g for 3 min. The upper aqueous phase containing RNA was carefully transferred to a new tube without disturbing the interface. RNA was precipitated by the addition of an equal volume of 70% ethanol and loaded onto a spin column from a PicoPure^™^ RNA Isolation kit (Arcturus, Mountain View, CA, USA) according to the manufacturer's instructions. RNA concentrations were determined using a NanoDrop ND-1000 spectrophotometer (NanoDrop Technologies Inc., Wilmington, USA). Total RNA (40 ng) was reverse-transcribed to produce cDNA using Multiscribe^™^ Reverse Transcriptase (Applied biosystems, Foster City, CA, USA) primed with random primers. In all cases, a reverse transcriptase negative control was used to evaluate genomic DNA contamination. The relative abundance of mRNA transcripts was calculated using the ΔΔcq method with the conserved helix-loop-helix ubiquitous kinase (*CHUK*) (Falco *et al*., 2006) as a reference gene on a Bio-Rad CFX Connect^™^ Real-Time PCR Detection System (Bio-Rad Laboratories Inc, Mississaugua, Canada) and QuantiFast SYBR Green PCR Master Mix (Qiagen, Toronto, Canada). The genes analyzed included *BAX*, *CYP51*, *DNMT3A*, *IGF2R*, *UBE2A*, *HSPA1A* and *MnSOD*, along with the reference gene. In each sample, cDNA was analyzed in quadruplicate to determine relative levels of each transcript of interest. In all cases, a reverse transcriptase negative control was included. The qRT-PCR reaction mix contained 12.5 μl 2× QuantiFast SYBR Green PCR Master Mix, 2μl (1875 nM) forward and reverse primers (Integrated DNA Technologies, Inc., IA, USA) specific for the genes of interest and 2 μl of cDNA template. The final volume was made up to 25 μl using nuclease-free water. Reactions were run at 95°C for 5 min followed by 40 cycles of 95°C for 10 s and 60°C for 1 min and a standard dissociation curve.

Primer sequences and approximate sizes of the amplified fragments of all transcripts are provided in Table 1. The efficiency of primer amplification was 90 to 100%. Non-template controls were not amplified or returned a Cq value 10 points higher than the average Cq for the genes. Expression levels of the target genes were normalized to average expression levels of the *CHUK* gene, which was expressed at constant levels (Cq values) in all samples and was stable under the conditions used.

**Table 1:** Primers used for reverse transcription–quantitative polymerase chain reaction.

Gene	Primer sequence	Fragment size	GenBank accession no.	Sequence references
*DNMT3A*	F: CCGTAGTGTCCAAGACCAATC R: GCTGAGGCAAATCCTCGTAAC	186	BC114063	[20]
*HSPA1A*	F: AAGGTG CTGGACAAGTGCCAGGAGGTGA R: ACTTGGAAGTAAACAGAAACG GGTGAAAA	503	U09861	[21]
*UBE2A*	F: GGGCTCCGTCTGAGAACAACATC R: CATACTCCCGCTTGTTCTCCTGG	336	XM_864331	[22]
*CYP51*	F: GCTCATTAGTTTGGGGGTGA R: TCCCCACCCATCCTTACATA	218	NM_001025319.2	[22]
*IGF2R*	F: CAGGTCTTGCAACTGGTGTATGA R: TTGTCCAGGGAGATCAGCATG	137	J03527	[23]
*BAX*	F: TCTGACGGCAACTTCAACTG R: TGGGTGTCCCAAAGTAGGAG	214	NM_173894.1	[24]
*MnSOD*	F: CCC ATGAAGCCCTTTCTAATCCTG R: TTCAGAGGCGCTACTATTTCCTTC	307	L22092.1	[21]
*CHUK*	F: TGATGGAATCTCTGGAACAGCG R: TGCTTACAGCCCAACAACTTGC	180	NM_174021.2	[25]

Abbreviations: F, forward; R, reverse

### Experimental design

#### Experiment 1

Characterize the effects of CLC added to a FCS-supplemented or chemically-defined medium before vitrification on *in vitro* matured bovine oocytes.

**Experiment 1a:** Live-cell imaging of cholesterol transport within *in vitro* matured bovine oocytes incubated with CLC in a FCS-supplemented or chemically-defined medium.

After 22 h of IVM, oocytes were denuded by gentle pipetting and incubated in 2 mg/ml BODIPY-labeled CLC at 38.5°C in HEPES-TCM-199 medium containing either 10% (v/v) FCS or 0.05% (w/v) PVA. BPY-Chol fluorescence was visualized in living oocytes at 30, 45, 60 and 75 min using a confocal microscope. A total of 20 oocytes were examined in each group in 3 different replicates.

**Experiment 1b:** Effects of CLC added to FCS-supplemented or chemically-defined medium before vitrification on the developmental competence of *in vitro* matured oocytes.

After 22 h of IVM and according to the results obtained in the previous experiment, partially denuded oocytes were randomly distributed into five groups: A and C) oocytes maintained in medium containing FCS; B) oocytes incubated in medium containing FCS with 2 mg/ml CLC; D) oocytes incubated in medium containing PVA with 2 mg/ml CLC; and E) oocytes incubated in medium containing PVA without CLC. Incubation with or without CLC was carried out at 38.5°C in 5% CO_2_ for 30 min. Oocytes were then washed 3 times in the corresponding handling medium without CLC. Oocytes were then vitrified/warmed as previously described using a FCS-supplemented medium (group A) or a synthetic medium containing 6% PVP and 1 mg/ml fetuin (groups B to E). After warming, oocytes were allowed to recover for 2 additional hours in IVM medium. Oocytes *in vitro* matured for 24 h were used as non-vitrified controls.

After 24 h of IVM, oocytes from each treatment group were *in vitro* fertilized and cultured. Cleavage rates were determined 48 hpi and blastocyst rates on Days 7 and 8 pi (8 replicates per group).

#### Experiment 2

Cholesterol tracking in living oocytes treated with different CLC concentrations and effects on embryo developmental competence after vitrification/warming

**Experiment 2a:** Live-cell imaging of cholesterol transport into *in vitro* matured bovine oocytes incubated with different CLC concentrations.

After 22 h in IVM, oocytes were denuded by gentle pipetting and incubated with 1 mg/ml, 2 mg/ml or 3 mg/ml BODIPY-labeled CLC in HEPES-buffered TCM-199 containing 10% (v/v) FCS at 38.5°C. BPY-Chol fluorescence was visualized in living oocytes using a confocal microscope at 10, 20, 30, 40 and 60 min. A total of 30 oocytes were examined in each group in 3 different replicates.

**Experiment 2b:** Effects of different CLC concentrations prior to vitrification on the developmental competence of *in vitro* matured bovine oocytes

After 22 h of *in vitro* maturation and according to the results obtained in Experiment 2a, oocytes were randomly distributed into 4 groups: Control Vit group = no CLC treatment; CLC 1 mg/ml 40’ group = oocytes incubated with 1 mg/ml CLC for 40 min; CLC 2 mg/ml 30’ group = oocytes incubated with 2 mg/ml CLC for 30 min; CLC 3 mg/ml 20’ group = oocytes incubated with 3 mg/ml CLC for 20 min. Oocytes were then washed 3 times in a medium without CLC and vitrified/warmed as previously described but using media supplemented with FCS. After warming, oocytes were allowed to recover for 2 additional hours in IVM medium. Oocytes *in vitro* matured for 24 h were used as non-vitrified controls.

After 24 h of IVM, oocytes from each treatment group were *in vitro* fertilized and cultured. Cleavage rates were determined 48 hpi and blastocyst rates on Days 7 and 8 pi (4 replicates per group).

#### Experiment 3

Effects of CLC treatment before vitrification of immature and *in vitro* matured oocytes on developmental competence and specific gene expression.

After follicle aspiration, a proportion of the COCs was randomly divided into the following groups: GV-VIT = vitrified GV oocytes; and GV-CLC-VIT = GV oocytes incubated with 2 mg/ml CLC for 30 min and vitrified. After vitrification, VIT group oocytes were warmed and transferred back to the maturation medium.

The rest of the COCs were matured *in vitro* for 22 h and randomly divided into three groups: Control = fresh non-vitrified MII oocytes; MII-VIT = vitrified MII oocytes; and MII-CLC-VIT = MII oocytes incubated with 2 mg/ml CLC for 30 min and vitrified. After vitrification, VIT group oocytes were warmed and transferred back to the maturation medium. After 24 h of IVM, oocytes from each treatment group were *in vitro* fertilized and cultured as previously described. In a first set of experiments (5 replicates), embryo development was allowed to continue until Day 5 when morulae from the different treatment groups were harvested for RNA extraction and RT-qPCR. In a second set of experiments, cleavage rates were determined 48 hpi and blastocyst rates on Days 7 and 8 pi (6 replicates per group)

### Statistical analysis

All statistical tests were performed using the software package SPSS version 21.0 for Windows (IBM Corp.; Chicago, Illinois, USA). Data are provided as the mean ± standard error of the mean (SEM) and significance was set at P≤ 0.05. The Shapiro-Wilk and Levene tests were used to check the normality of data and homogeneity of variances, respectively.

On the one hand, the effects of CLC, handling medium and vitrification medium on the development competence of *in vitro* matured and fertilized bovine oocytes were tested through a three-way analysis of variance followed by post-hoc Sidak’s test. The effects of CLC treatment of *in vitro* matured bovine oocytes in an FCS-supplemented or chemically-defined medium before vitrification on the developmental competence of embryos derived from these oocytes were evaluated through one-way ANOVA, followed by post-hoc Sidak’s test. Embryo developmental competence of immature and *in vitro*-matured bovine oocytes pretreated with CLC before vitrification was evaluated through a two-way ANOVA (factors: stage and treatment) followed by post-hoc Sidak’s test.

With regard to analysis of gene expression, relative expression of six out of seven genes (all except *CYP51*) did not match with parametric assumptions. For this reason, we attempted to transform our data through square root (√x) or arcsin square root (arcsin √x). Transformation was only successful in the case of arcsin √(*IGF2R*). Therefore, the effects of treatment (i.e. fresh, vitrified-warmed control, vitrified-warmed + CLC) and stage (GV, MII) upon the expression of *CYP51* and *IGF2R* were tested through a two-way analysis of variance (ANOVA) followed by Sidak’s test for pair-wise comparisons. For the relative transcript abundance of the other genes (*DNMT3*, *HSPA1A*, *MnSOD*, *BAX* and *UBE2A*), we performed a non-parametric Scheirer-Ray-Hare ANOVA for ranked data (again, factors were treatment and stage). Following calculation of the ‘H’ statistic, the Mann-Whitney test was run for pair-wise comparisons.

## Results

### Experiment 1

Characterize the effects of CLC added to a FCS-supplemented or chemically-defined medium before vitrification on *in vitro* matured bovine oocytes.

#### Experiment 1a

Live-cell imaging of cholesterol transport within *in vitro* matured bovine oocytes incubated with CLC in a FCS-supplemented or chemically-defined medium.

**Fig 1:**
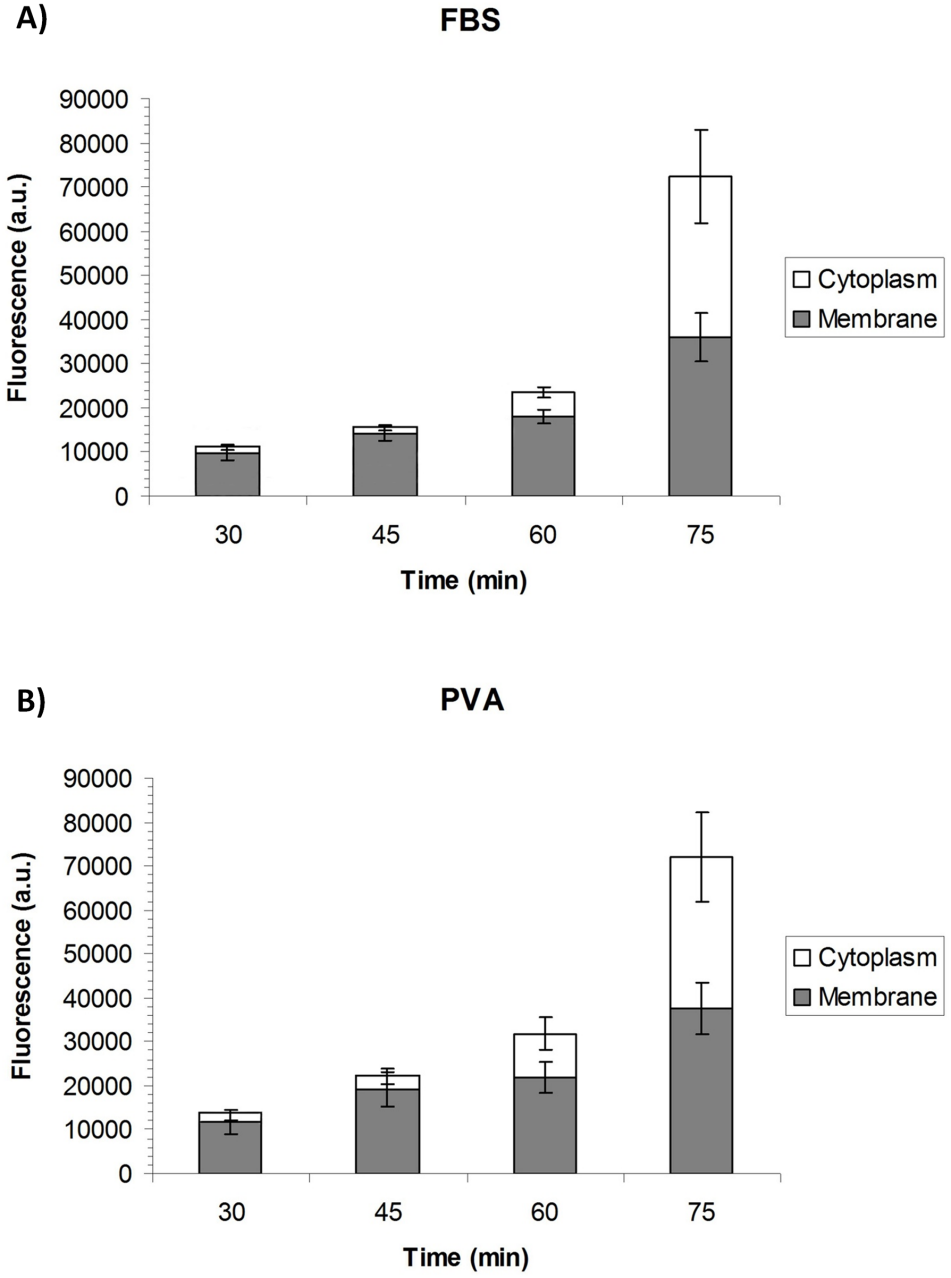
Subcellular localization of BPY-Chol in *in vitro* matured bovine oocytes. Fluorescence intensity observed at the membrane and in the cytoplasm of bovine oocytes treated with 2 mg/ml CLC in a medium containing FCS or PVA at different chase times: 30, 45, 60 and 75 minutes. Mean ± SEM values for each subcellular compartment over time.

To examine whether cholesterol in a medium supplemented with FCS was transported into the oocyte, cholesterol internalization at different chase times was imaged in living bovine oocytes incubated with 2 mg/ml CLC in a medium containing FCS (Fig 1A) or PVA (Fig 1B). After 30 min of incubation, the plasma membrane showed a high labeling intensity relative to the cytoplasm. As chase time lengthened, cholesterol inclusion in the cytoplasm also increased considerably. After 75 min, cholesterol trafficking through the plasma membrane into the cytoplasm showed the same pattern when the medium was supplemented with FCS or with PVA.

#### Experiment 1b

Effects of CLC added to FCS-supplemented or chemically-defined medium before vitrification on the developmental competence of IVM oocytes

Given that cholesterol labeling could be observed at the plasma membrane after 30 min of incubation in the previous experiments, *in vitro* matured oocytes were vitrified/warmed following pre-treatment with 2 mg/ml CLC for 30 min in a FCS- or PVA-supplemented medium. The vitrification medium used for this experiment was supplemented with PVP and fetuin to avoid the use of FCS during the vitrification process. Table 2 shows the results of oocyte survival and embryo development after oocytes were subjected to each treatment. Significantly higher survival, cleavage and blastocyst rates were observed for non-vitrified oocytes than vitrified oocytes regardless of CLC treatment or the holding medium used. When comparing the vitrification groups, oocytes from group C, held in FCS-supplemented medium before vitrification in synthetic medium showed a significantly lower survival rate compared to the other vitrification groups. Oocytes from group C also showed significantly lower cleavage rates when compared to the group of oocytes held and vitrified in media containing FCS (group A) while no significant differences were observed for oocytes in groups B, D and E when compared to groups A and C. No significant differences in Day 7-blastocyst rates were observed among the vitrification groups. However, oocytes from group D showed significantly lower Day 8-blastocysts rate than oocytes from group A. When evaluating embryo expansion as an indicator of embryo quality, CLC pretreatment of oocytes in the presence of PVA (group D) resulted in significantly reduced expansion rates on Day 8 pi when compared to those recorded in fresh non-vitrified oocytes, while the remaining vitrification groups showed similar rates of expansion to the control and D groups. Only embryos derived from oocytes from group A (held and vitrified in media supplemented with FCS) were able to hatch, although no significant differences in rates were observed between this group and the remaining vitrification groups.

**Table 2:** Effect of CLC treatment of *in vitro* matured bovine oocytes in an FCS-supplemented or chemically-defined medium before vitrification on the developmental competence of embryos derived from these oocytes.

	CLC	Handling medium	Vit medium	n	Mean % ± SEM				Day 8 embryo Mean % ± SEM		
					Survival	Cleavage	Blastocyst Day 7	Blastocyst Day 8	Non-expanded	Expanded	Hatched
**Control**	-	FCS	-	552	95.7 ± 1.6 ^a^	75.1 ± 2.6 ^a^	19.8 ± 1.9 ^a^	22.0 ± 1.8 ^a^	21.3 ± 4.2 ^a^	58.8 ± 5.0 ^a^	19.9 ± 4.8^a^
**A**	-	FCS	FCS	383	71.6 ± 3.2 ^b^	44.4 ± 2.7 ^b^	4.3 ± 1.0 ^b^	7.2 ±1.0 ^b^	64.9 ± 10.0 ^b^	31.5 ± 9.5 ^a^^b^	3.6 ± 3.6 ^b^
**B**	+	FCS	PVP+ F	121	60.6 ± 12.3 ^b^	31.5 ± 8.5 ^b^^c^	3.1 ± 1.3 ^b^	5.5 ± 2.5 ^b^^c^	33.3 ± 19.2 ^a^^b^	66.7 ± 19.2 ^a^^b^	0 ^b^
**C**	-	FCS	PVP+ F	109	49.7 ± 2.8 ^c^	24.8 ± 3.7 ^c^	2.6 ± 1.7^b^	4.6 ± 2.2 ^b^^c^	55.6 ± 29.4 ^a^^b^	44.4 ± 29.4 ^a^^b^	0 ^b^
**D**	+	PVA	PVP+ F	186	66.0 ± 5.5 ^b^	37.4 ± 4.0 ^b^^c^	2.6 ± 1.0 ^b^	3.5 ± 1.0 ^c^	90.0± 10.0^b^	10.0±10.0 ^b^	0 ^b^
**E**	-	PVA	PVP+ F	293	64.0± 5.3 ^b^	34.6 ± 3.1 ^b^^c^	2.7 ± 0.7 ^b^	5.9 ± 0.9 ^b^^c^	68.5± 10.2^b^	31.5±10.2 ^a^^b^	0 ^b^

CLC treatment: oocytes were incubated with 2 mg/ml CLC for 30 min. Handling medium: the handling medium used during CLC incubation contained PVA or FCS. Vit medium: vitrification/warming medium contained FCS or PVP+Fetuin. Data are provided as the mean ± SEM (standard error of the mean).

^a^ Values with different subscripts within columns differ significantly (*P<*0.05).

^b^ Values with different subscripts within columns differ significantly (*P<*0.05).

^c^ Values with different subscripts within columns differ significantly (*P<*0.05).

### Experiment 2

Cholesterol tracking in living bovine oocytes treated with different CLC concentrations and effect on embryo development competence after vitrification/warming

#### Experiment 2a

Live-cell imaging of cholesterol transport into *in vitro* matured bovine oocytes incubated with different CLC concentrations.

**Fig 2:**
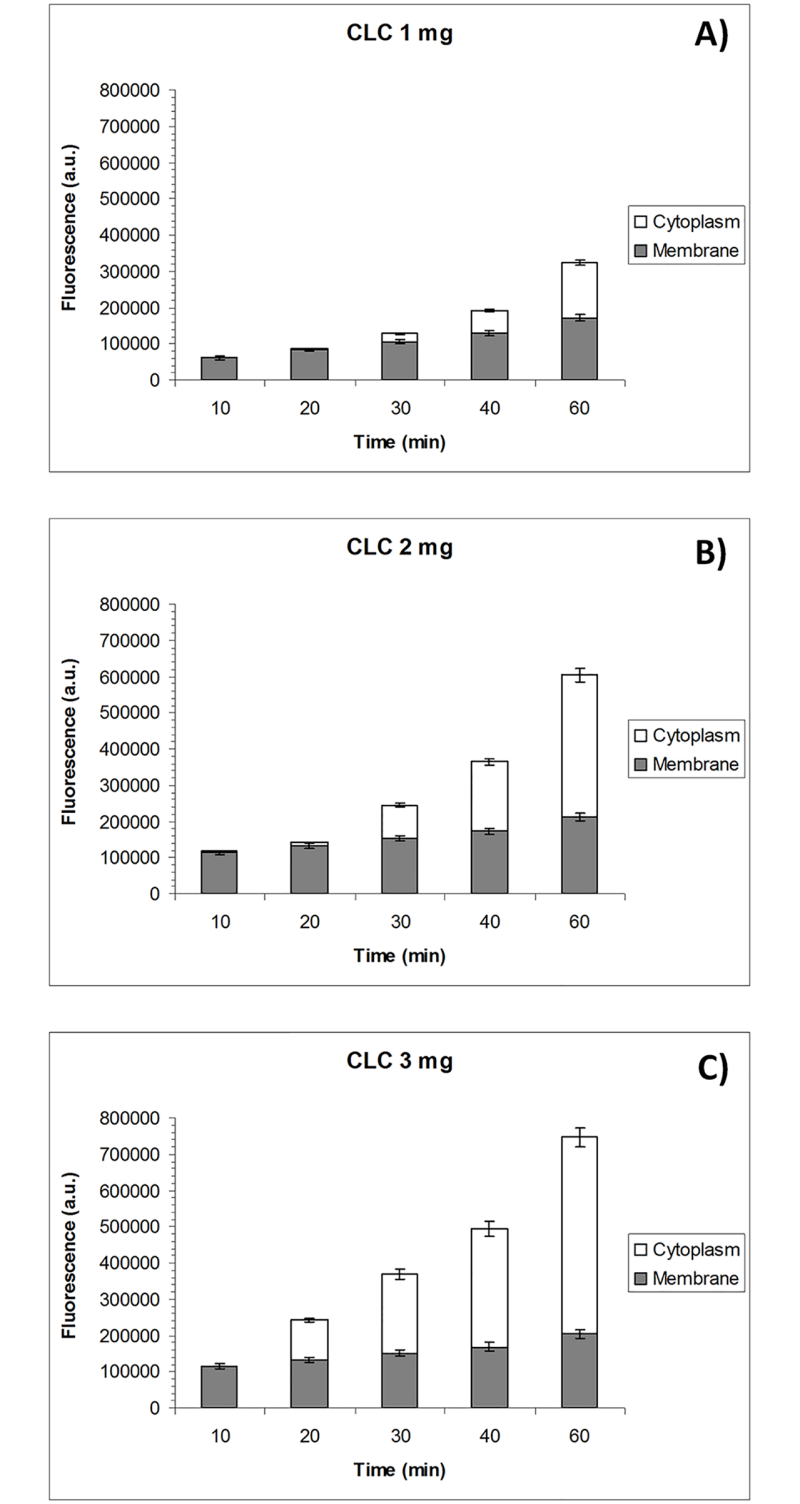
Subcellular localization of BPY-Chol in *in vitro* matured bovine oocytes incubated with CLC. Fluorescence intensity observed at the plasma membrane and in the cytoplasm of bovine oocytes treated with 1, 2 or 3 mg/ml CLC at different chase times: 10, 20, 30, 40 and 60 minutes. Mean ± SEM values for each subcellular compartment over time.

**Fig 3:**
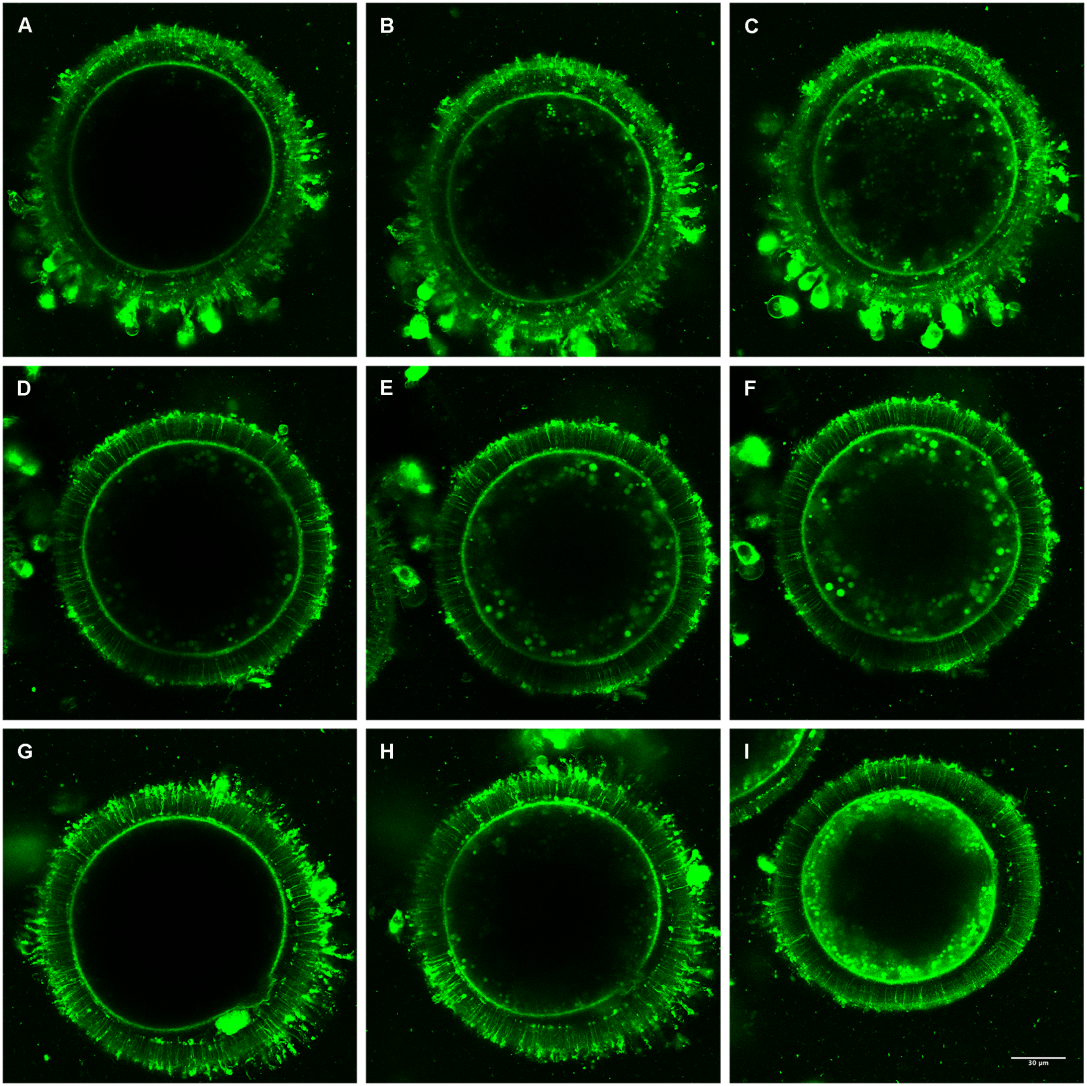
Immunofluorescence localization of BODIPY-cholesterol in *in vitro* matured oocytes. The plasma membrane and cytoplasm localization of cholesterol was detected using the BODIPY fluorescent probe in *in vitro* matured bovine oocytes incubated with different concentrations of CLC. (A-C) oocytes incubated with 1 mg/ml of CLC imaged at 20, 40 and 60 minutes; (D-F) oocytes incubated with 2 mg/ml of CLC imaged at 30, 40 and 60 minutes; (G-I) oocytes incubated with 3 mg/ml of CLC imaged at 10, 20 and 40 minutes. BPY-Chol could be detected only at the plasma membrane (A, G), at the plasma membrane and diffusing into the cytoplasm (B,D,H) or at the plasma membrane and mostly distributed in the cytoplasm (E,C,F,I). Fluorescence intensity was quantified using ImageJ software. Scale bar = 30 μm.

Internalization of fluorescence-labeled cholesterol was followed by confocal microscopy imaging of living bovine oocytes incubated with 1 mg/ml (Fig 2A), 2 mg/ml (Fig 2B) or 3 mg/ml CLC (Fig 2C) at different chase times. As in the previous experiment, total cell fluorescence increased with incubation time. When oocytes incubated with 2 mg/ml or 3 mg/ml CLC were imaged after 10 min, intense labeling was observed at the plasma membrane while there was essentially no labeling in the cytoplasm. This pattern was repeated when the incubation time was extended to 20 min in the presence of 1 mg/ml CLC. For the highest CLC concentration (3 mg/ml), fluorescence could be observed diffusing into the cytoplasm as early as 20 min, while for 1 mg/ml CLC and 2 mg/ml CLC, 40 and 30 min respectively were required before fluorescence spread into the cytoplasm. Fluorescence intensity in the cytoplasm increased with incubation time and higher levels of fluorescence were observed for oocytes incubated with the highest CLC concentrations (2 and 3 mg/ml). Images of cholesterol internalization into the oocytes are provided in Fig 3.

#### Experiment 2b

Effects of different CLC concentrations prior to vitrification on the developmental competence of *in vitro* matured bovine oocytes.

To select the incubation time and CLC concentration for this experiment we looked for high fluorescence intensity at the plasma membrane level along with some scattered fluorescence in the cytoplasm (<25%). This level of fluorescence indicated that the plasma membrane contained relatively high amount of CLC and that the low diffusion of CLC into the cytoplasm would not be likely to impair oocyte survival after vitrification. Thus, based on the results obtained in Experiment 2a and these fluorescence criteria, we tested different incubation times and concentrations of CLC prior to vitrification of *in vitro* matured oocytes to assess their effects on early *in vitro* embryo development (Table 3). While differences among treatments were not observed in terms of survival rates, cleavage rates were significantly reduced in the groups of oocytes treated with 1 mg/m CLC for 20 min or 2 mg/ml for 30 min when compared with control fresh oocytes, while this was not observed in the remaining vitrification groups. Day 7 and Day 8 blastocyst yields were significantly higher for fresh non-vitrified oocytes when compared to the vitrified oocytes. Among the vitrification groups, no significant differences in Day 7 and Day 8 blastocyst yields were observed, regardless of the concentration or incubation time.

**Table 3:** Comparison of different incubation times and concentrations of CLC prior to vitrification on the developmental competence of *in vitro*-matured bovine oocytes.

Treatment	n	Mean % ± SEM				Day 8 embryos Mean % ± SEM		
		Survival	Cleavage	Blastocysts Day 7	Blastocysts Day 8	Non-expanded	Expanded	Hatched
**Control**	191	91.9 ± 5.7	62.4 ± 4.7 ^a^	15.9 ± 1.6 ^a^	18.7 ± 0.1^a^	36.0 ± 22.4^a^	50.4 ± 16.8	13.6 ± 8.2
**Control Vit**	101	80.2 ± 0.2	48.4 ± 6.7 ^a^^b^	7.1 ± 1.4^b^	8.8 ± 1.1^b^	33.3 ± 19.2^a^	66.7 ±19.2	0
**CLC 1 mg/ml 40’**	107	80.5 ± 5.3	33.6 ± 1.9 ^b^	2.0 ± 1.2^b^	5.0 ± 1.4^b^	75.0 ± 25.0^b^	25.0 ± 25.0	0
**CLC 2 mg/ml 30’**	114	80.5 ± 4.9	37.1 ± 6.8 ^b^	5.7 ± 3.2^b^	8.4 ± 1.8^b^	63.9 ± 21.7^a^^,^ ^c^	36.1 ± 21.7	0
**CLC 3 mg/ml 20’**	101	80.8 ± 13.7	51.0 ± 13.0 ^a^^b^	6.0 ± 2.2^b^	7.4 ± 2.0^b^	72.2 ± 14.7^b^^,^ ^c^	27.8 ±14.7	0

Treatments: Control = oocytes *in vitro* matured for 24 h; Control Vit: no CLC treatment before vitrification; CLC 1 mg/ml 40’ = oocytes incubated with 1mg/ml CLC for 40 min before vitrification; CLC 2 mg/ml 30’ = oocytes incubated with 2 mg/ml CLC for 30 min before vitrification; CLC 3 mg/ml 20’ = oocytes incubated with 3 mg/ml CLC for 20 min before vitrification. Data are provided as the mean ± SEM.

^a^ Values with different subscripts within columns differ significantly (*P<*0.05).

^b^ Values with different subscripts within columns differ significantly (*P<*0.05).

^c^ Values with different subscripts within columns differ significantly (*P<*0.05).

### Experiment 3

Effects of CLC treatment before vitrification of immature and *in vitro* matured oocytes on developmental competence and specific gene expression.

Table 4 shows the *in vitro* embryo developmental competence of oocytes –immature or *in vitro*-matured—pretreated with 2 mg/ml CLC for 30 min prior to vitrification. All vitrification treatments led to significantly lower percentages of embryo cleavage and Day 7 or Day 8 blastocyst yields (P<0.05) compared to fresh non-vitrified oocytes. Cleavage and blastocyst rates were similar for all vitrified/warmed oocytes, regardless of CLC treatment or nuclear stage. Furthermore, all vitrified oocyte groups returned similar rates of blastocyst expansion to the control fresh oocyte group except for a significantly higher percentage observed for the vitrified MII oocytes. Only matured MII oocytes treated with CLC before vitrification also demonstrated similar percentages of blastocyst hatching to control fresh oocytes, although rates did not differ statistically from those observed in the other vitrification groups.

**Table 4:** Embryo developmental competence of immature and *in vitro*-matured bovine oocytes pretreated with CLC before vitrification.

Treatment	n	Mean % ± SEM				Day 8 embryos Mean % ± SEM		
		Survival	Cleavage	Blastocyst Day 7	Blastocyst Day 8	Non-expanded	Expanded	Hatched
**Control**	185	93.4 ± 4.3^a^	66.7 ± 3.6^a^	18.4 ± 1.6 ^a^	19.4 ± 0.8 ^a^	42.6 ± 10.9 ^a^	35.6 ±10.2 ^a^	21.8 ± 10.0 ^a^
**GV-VIT**	238	72.0 ± 7.2^a^^b^	41.4 ±5.5^b^	5.6 ± 1.1^b^	7.3 ± 0.8 ^b^	66.7 ±16.7^a^^b^	27.8 ± 12.7 ^a^	5.6 ± 5.6 ^b^
**GV- CLC-VIT**	236	64.0 ± 4.9 ^b^	40.9 ± 4.8^b^	5.8 ± 0.7^b^	5.7 ± 1.5 ^b^	75.00 ±11.2^b^	25.0 ±11.2 ^a^	0.0 ± 0.0 ^b^
**MII-VIT**	180	60.8 ± 10.0 ^b^	39.9 ± 5.5^b^	7.3 ±2.1^b^	7.0 ± 1.3 ^b^	38.9 ± 15.3 ^a^	61.1 ± 15.3 ^b^	0.0 ± 0.0^b^
**MII-CLC-VIT**	206	69.0 ± 5.1 ^b^	41.7 ±2.2^b^	8.9 ± 1.3^b^	9.9 ± 1.7 ^b^	36.1 ± 15.8 ^a^	55.6 ±13.4 ^a^^b^	8.3 ± 5.7^a^^b^

Treatments: Control = fresh non-vitrified MII oocytes; GV VIT = vitrified GV oocytes; GV-CLC-VIT = GV oocytes vitrified after 30 min incubation with 2 mg/ml CLC; MII VIT = vitrified MII oocytes; MII-CLC-VIT = MII oocytes vitrified after 30 min incubation with 2 mg/ml CLC. Data are provided as the mean ± SEM.

^a^ Values with different subscripts within columns differ significantly (*P<*0.05).

^b^ Values with different subscripts within columns differ significantly (*P<*0.05).

^c^ Values with different subscripts within columns differ significantly (*P<*0.05).

**Fig 4:**
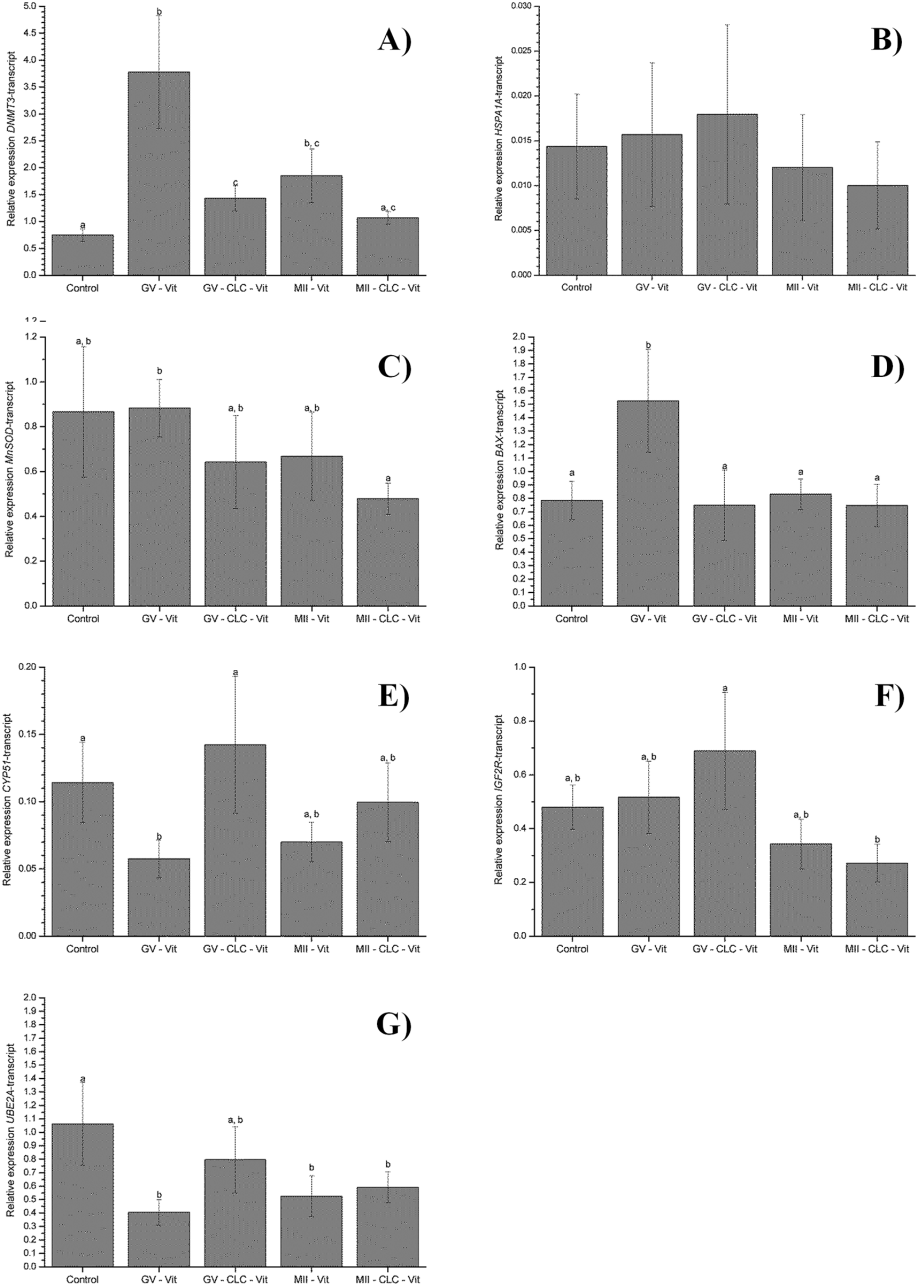
Relative expression levels of the genes *DNMT3* (A), *HSPA1A* (B), *MnSOD* (C), *BAX* (D), *CYP51* (E), *IGF2R (F)*, *UBE2A* (G) recorded in *in vitro* produced morulae. GV = oocytes at the germinal vesicle stage; MII = oocytes at the metaphase II stage; Control = fresh, non-vitrified oocytes; Vit = vitrified oocytes; CLC +Vit = Oocytes pre-treated with 2 mg/ml CLC for 30 min and vitrified. Data are provided as the mean ± SEM. Different subscripts indicate significant differences (*P*<0.05) in gene expression.

In order to determine whether changes in the expression of developmentally-important genes were associated with the different vitrification protocols, we next evaluated the levels of *DNMT3A*, *HSPA1A*, *MnSOD*, *BAX*, *CYP51*, *IGF2R*, *UBE2A* mRNAs *in vitro* produced morulae (Fig 4). No significant differences were detected in relative mRNA abundances for *HSPA1A*, *MnSOD* and *IGF2R* in morulae derived from GV vitrified oocytes or MII vitrified oocytes compared to non-vitrified oocytes, regardless of CLC treatment. *DNMT3A* and *BAX* gene expression was significantly higher in morulae from vitrified GV oocytes. The expression of both genes in CLC-treated vitrified GV oocytes remained similar when compared to the levels observed in non-vitrified fresh oocytes. In contrast, *CYP51* expression was significantly lower in morulae from vitrified GV oocytes compared to morulae from CLC-treated vitrified or non-vitrified oocytes. While the expression of the *BAX* gene in vitrified MII oocytes remained similar to that in the fresh oocyte control group regardless of CLC treatment, *DNMT3A* expression was significantly higher in morulae from vitrified MII oocytes, and no differences were observed in morulae from oocytes vitrified after CLC treatment. *UBE2A* gene expression was significantly lower in morulae from GV- or MII vitrified or MII-CLC-vitrified oocytes, while expression levels of this gene in morulae derived from GV-CLC-vitrified oocytes remained similar to those recorded in control fresh oocytes.

## Discussion

Cryopreserved oocytes show highly compromised survival and developmental capacities due to the morphological and cytological damage induced by the cryopreservation process. Cells with more flexible membranes that are more permeable to water and intracellular cryoprotectants are known to incur less damage during cryopreservation [26]. Adding cholesterol to the incubation medium increases membrane fluidity at lower temperatures by modifying how membrane phospholipids interact with each other, making membranes less contracted at lower temperatures [27].

Horvath and Seidel [8] detected no effects of pre-treatment of *in vitro* mature bovine oocytes with CLC in the presence of FCS before vitrification on subsequent embryo developmental competence. In contrast, when a synthetic medium was used during CLC treatment (2 mg/ml CLC for 1 h), cleavage and development up to eight-cell embryo rates were significantly higher for oocytes pretreated with CLC prior to vitrification. These authors attributed the lack of effect of CLC treatment in improving cryotolerance that cholesterol was preferentially transferred to lipids or proteins present in the fetal calf serum (FCS) avoiding its incorporation into the oocyte [8]. In the present study, through in vivo confocal microscopy and BPY-Chol labeling, we observed cholesterol trafficking into oocytes at a specific CLC concentration (2 mg/ml) using a handling medium supplemented either with FCS or PVA. Moreover, in both the FCS and PVA supplemented media, CLC was observed at the plasma membrane 30 min after incubation. Based on these results, we compared the effect on embryo development of CLC treatment (2 mg/ml for 30 min) prior to vitrification using the two supplements. Our results showed that neither prior CLC treatment, nor the use of FCS or PVA, conferred any benefits in terms of embryo development up to the blastocyst stage. It has been shown that both cholesterol depletion and cholesterol enrichment of the plasma membrane may affect cellular functions [28]. In our study, we postulate that any improvements attributable to plasma membrane cholesterol enrichment may have been offset by cell alterations resulting from changes in phospholipid distributions and modified cholesterol distribution among the cell compartments due to the presence of CLC [28–30]. This may have had an effect on the oocyte plasma membrane and on the subsequent developmental competence of the oocyte after vitrification.

The results obtained in this study for the use of FCS or synthetic macromolecular components in the vitrification solution are comparable to those of other studies examining small volume supports for vitrification [17, 31, 32]. The solutions used for oocyte *in vitro* maturation, handling, vitrification or warming usually contain FCS [17, 31, 33–35]. Since we observed no improvement in embryo developmental competence after the vitrification of oocytes handled or vitrified in synthetic media, FCS was used as a supplement for the rest of our experiments. It has been already reported that the efficiency of cholesterol transfer from βCD inclusion complexes to biological membranes depends on βCD-cholesterol concentration and exposure duration [5, 36, 37]. Because no differences in embryo development were observed here after incubation with 2mg/ml CLC for 30 min we used confocal microscopy to identify the time-point at which fluorescent-tagged cholesterol was primarily located at the plasma membrane using three different concentrations of CLC: 40 min for 1 mg/ml, 30 min for 2 mg/ml and 20 min for 3 mg/ml. Cholesterol manipulation as carried out in this study may have modified the plasma membrane properties of matured oocytes pre-treated with different CLC concentrations for varying time intervals. However, we observed no correlation between oocyte cryotolerance and embryo cleavage or embryo development up to the blastocyst stage. The effects of cholesterol on membranes depend on lipid composition and fatty acid saturation. Cholesterol and sphingomyelin are thought to form ordered lipid domains (rafts) in mammalian cell membranes [28]. Cholesterol disrupts the highly ordered gel phase of brain sphingomyelin, leading to a more fluid membrane, while reducing the fluidity of brain phosphatidylcholine bilayers [38]. We propose that the irregular incorporation of labeled lipids into the different cholesterol domains of the oocyte plasma membrane may preclude increases in membrane fluidity in response to the addition of cholesterol.

Cryobiological differences between immature and *in vitro* matured oocytes prompted our subsequent studies in which oocytes at the GV or MII stage were pretreated with 2 mg/ml CLC for 30 min before vitrification, after which the impact on embryo development and gene expression was determined. Although no differences in cleavage and blastocyst yields were observed after CLC treatment, differences were detected in the relative abundance of some gene transcripts during early embryo development. For example, the relative abundance of *DNMT3A* transcripts in morulae derived from immature and *in vitro* matured vitrified oocytes was significantly higher compared to abundances observed in morulae derived from fresh oocytes. Similarly, Shirazi et al. [39] found that the overall expression of *DNMT3B* in morulae derived from vitrified immature ovine oocytes was greater than in morulae derived from non-vitrified fresh oocytes. *DNMT3A* and *DNMT3B* drive most *de novo* methylation during embryonic reprogramming whereby methyl groups are added to previously unmodified DNA [40]. DNA methylation exerts essential functions in many genetic processes such as X chromosome inactivation, genome imprinting, and transcriptional silencing of specific genes and repetitive elements [41, 42]. Given its role in de novo methylation, abnormal levels of *DNMT3A* expression could suggest that embryos derived from vitrified oocytes are at higher risk for problems in these processes, which could be ameliorated by CLC treatment prior to vitrification. Importantly, CLC treatment prior to vitrification of both GV and MII bovine oocytes gave rise to morulae showing levels of *DNMT3A* transcripts approaching those of morulae derived from non-vitrified oocytes. Similarly, when the expression of *UBE2A*, another gene related to developmental epigenetics, was analyzed, morulae derived from GV vitrified after CLC treatment showed similar expression levels to those derived from non-vitrified oocytes. Further work is needed to assess global levels of DNA methylation and histone acetylation in oocytes pretreated with CLC prior to vitrification.

The expression of *BAX* was also significantly higher in morulae derived from vitrified GV oocytes than morulae derived from fresh or CLC-treated GV oocytes. When *BAX* is overexpressed in cells, apoptotic death is accelerated and previous research has shown that morphologically poor quality or fragmented embryos show higher expression levels of this gene [43, 44]. In response to pre-treatment with CLC before vitrification of GV oocytes, *BAX* gene expression levels returned to similar levels to those observed in fresh control oocytes. This suggests that CLC may have helped improve the quality of embryos obtained after oocyte vitrification. *CYP51* (cytochrome P450 family 51 or lanosterol 14a-demethylase *CYP51*) is required for de novo cholesterol synthesis during embryogenesis, which is essential for the regulation of membrane fluidity and thereby related to survival after embryo vitrification. Because blastocyst quality correlates positively with embryo survival after vitrification [45, 46], CLC treatment before vitrification of GV oocytes may also have served to improve embryo quality after vitrification of GV oocytes. While *CYP51* gene expression was significantly lower in morulae derived from vitrified GV oocytes, morulae derived from GV-CLC vitrified oocytes showed similar relative abundances of mRNA transcripts to morulae derived from fresh non-vitrified oocytes.

Embryos also show changes in expression of genes reflecting the stress response to suboptimal conditions [21]. Differences in gene transcript levels between morulae derived from vitrified oocytes treated with CLC compared to those without were higher for immature oocytes. This suggests that vitrification at the GV stage may be particularly harmful for the oocyte and that treatment with 2 mg/ml CLC for 30 min may protect the oocyte from specific stresses, giving rise to pre-embryos of higher developmental potential. Even though differences in developmental competence in terms of blastocyst rates were not observed after CLC treatment, differences in the relative abundances of some gene transcripts important for early embryo development were detected. Differences in the effects of CLC treatment between GV and MII oocytes might be related to their different cryotolerances,. For example, hydraulic conductivity is 2-fold lower in GV oocytes compared to MII oocytes [47, 48]. Thus, MII stage bovine oocytes have higher water and CPA permeability coefficients than GV stage oocytes [47, 48]. This implies that GV oocytes are less able to tolerate water flux or shear force related damage during volume changes [49]. Accordingly, GV oocytes may benifit more from membrane cholesterol enrichment through CLC treatment. Since membranes are stabilized by cholesterol and remain more fluid at lower temperatures, stresses associated with membrane distortion due to dehydration and shrinkage during vitrification, especially damaging for GV stage oocytes, are likely to be be reduced [50].

In conclusion, CLC pre-treatment before the vitrification of bovine *in vitro* matured oocytes does not appear to affect subsequent cleavage and embryo development rates, irrespective of the presence of FCS in the handling medium, the CLC concentration, incubation time or meiotic oocyte stage (GV or MII). However, relative differences in the expression of genes related to embryo development in oocytes pretreated with 2 mg/ml suggests that CLC does improve some aspects of embryo quality after vitrification, particularly when derived from oocytes vitrified at the GV stage. Further studies will obviously be necessary to determine whether the changes in gene expression that we have identified, and the processes in which they participate, can be further enhanced to optimize oocyte quality after vitrification.
